# Regulatory Effect and Mechanism of Tanshinone I on Cell Apoptosis in Steroid‐Induced Osteonecrosis of the Femoral Head

**DOI:** 10.1002/kjm2.70086

**Published:** 2025-09-18

**Authors:** Qing Su, Jia Chen

**Affiliations:** ^1^ Department of Bone Oncology Yantai Shan Hospital Yantai Shandong Province China; ^2^ Department of Rheumatology Immunology Shaoyang Central Hospital Shaoyang Hunan Province China

**Keywords:** osteocytes, phosphatidylinositol 3‐kinase/AKT/mammalian target of rapamycin, phosphorylation, steroid‐induced osteonecrosis of the femoral head, Tanshinone I

## Abstract

Steroid‐induced osteonecrosis of the femoral head (SIONFH) is a debilitating orthopedic condition. This study investigated the mechanism of Tanshinone I (TsI) in SIONFH modulating apoptosis in SIONFH via the PI3K/AKT/mTOR pathway. A SIONFH rat model was treated with TsI and a PI3K activator. Bone mineral density (BMD), bone volume/total volume (BV/TV), trabecular number (Tb.N), trabecular thickness (Tb.Th), and trabecular separation (Tb.Sp) were determined by microCT. Empty lacunae count, Osteopontin, and apoptosis of the femoral head tissues were assessed. Levels of Bax, cleaved‐caspase‐3, Bcl‐2, and AKT, PI3K, and mTOR phosphorylation in femoral head tissues were determined by Western blot. SIONFH rats exhibited decreased BMD, BV/TV, Tb.N, and Tb.Th, increased Tb.Sp, reduced Osteopontin‐positive cells, increased empty lacunae rate, and TUNEL and Osteopontin co‐positive cells, elevated Bax and cleaved‐caspase‐3 protein levels, and diminished Bcl‐2 protein expression. TsI promoted osteogenesis, attenuated SIONFH, and reduced apoptosis in SIONFH rats. TsI inhibited AKT, PI3K, and mTOR phosphorylation levels in the femoral head tissues of SIONFH rats, thereby repressing the PI3K/AKT/mTOR pathway activation. Activating the PI3K/AKT/mTOR pathway partially reversed TsI's effect in rats. Collectively, TsI limited the PI3K/AKT/mTOR pathway activation to reduce osteocyte apoptosis in SIONFH rats, which provided potential therapeutic insights for SIONFH treatment.

## 1 Introduction

Osteonecrosis of the femoral head (ONFH) is a debilitating orthopedic disorder characterized by structural deterioration, subchondral collapse of the femoral head, and progressive joint destruction [1]. Although ONFH is often asymptomatic in the early stages, it can eventually manifest as hip pain and restricted joint mobility upon physical examination [2]. ONFH is generally classified into traumatic and non‐traumatic forms, with non‐traumatic ONFH being associated with risk factors such as excessive alcohol consumption, autoimmune diseases, and, notably, prolonged corticosteroid use [3]. Among these, steroid‐induced ONFH (SIONFH) is increasingly prevalent, particularly among young and middle‐aged adults, and is recognized as one of the most common non‐traumatic causes of ONFH [4]. The condition is closely linked to high‐dose or long‐term corticosteroid administration and has emerged as a growing public health concern worldwide due to its increasing incidence and poor prognosis [2].

Tanshinone I (TsI), a bioactive diterpenoid compound extracted from *Salvia miltiorrhiza* (Danshen), has garnered interest for its therapeutic potential in skeletal disorders, including SIONFH [5, 6]. TsI exhibits anti‐inflammatory and anti‐resorptive properties, notably by suppressing osteoclast differentiation and reducing the formation of multinucleated osteoclasts [7]. Furthermore, TsI has demonstrated protective effects against lipopolysaccharide (LPS)‐induced alveolar bone loss in rats, suggesting its potential for mitigating bone degradation in inflammatory conditions [8]. These findings point to the therapeutic promise of TsI in preventing or attenuating osteonecrosis, although the molecular mechanisms underpinning its protective role remain to be fully elucidated.

Apoptosis is a tightly regulated, genetically programmed form of cell death that plays a fundamental role in maintaining cellular homeostasis and is essential for bone development, remodeling, and repair [9]. It can be initiated through several key pathways, most notably via activation of death receptors from the tumor necrosis factor (TNF) receptor superfamily, as well as mitochondria‐ and endoplasmic reticulum‐mediated mechanisms [10]. Emerging evidence suggests that TsI exerts anti‐apoptotic effects in various biological contexts. For instance, TsI has been reported to protect endothelial cell function by preserving angiogenic properties, such as tube formation and migration in human umbilical vein cells (EA.hy926) [11]. In addition, TsI has been shown to inhibit extracellular matrix degradation, inflammation, and chondrocyte apoptosis, thereby alleviating osteoarthritis in murine models [12].

Several phytochemicals have demonstrated therapeutic efficacy in SIONFH by modulating apoptosis. For example, Gastrodin reduces osteocyte apoptosis by downregulating Bax/Bcl‐2 ratios and suppressing caspase‐3 expression [13]. Astragaloside IV alleviates SIONFH symptoms by repolarizing M1 pro‐inflammatory macrophages toward the anti‐inflammatory M2 phenotype, thereby decreasing TNF‐α and IL‐1β levels and promoting osteocyte survival [14]. Echinacoside has also been shown to improve bone microarchitecture and reduce osteoblast apoptosis in SIONFH through modulation of the PI3K/AKT/FOXO1 pathway [15]. While these findings underscore the role of apoptosis in the pathophysiology of SIONFH, the precise effects of TsI on apoptosis in this context remain to be elucidated.

The phosphatidylinositol 3‐kinase (PI3K)/AKT/mammalian target of rapamycin (mTOR) signaling axis is a critical regulator of cellular processes such as metabolism, survival, inflammation, and apoptosis [16]. Modulating this pathway has been shown to promote osteogenic differentiation and suppress apoptosis in osteoblasts [17], and it plays a pivotal role in skeletal formation both in vitro and in vivo [18]. Recent studies have revealed that inhibition of PI3K/AKT/mTOR signaling can attenuate glucocorticoid (GC)‐induced apoptosis in bone cells [19]. Moreover, miR‐27a enhances osteogenic differentiation in GC‐treated human bone marrow mesenchymal stem cells by targeting this same pathway [20]. Notably, TsI has been implicated in regulating both autophagy and apoptosis by suppressing PI3K/AKT/mTOR pathway activation [21]. However, whether TsI exerts anti‐apoptotic effects in SIONFH via this signaling cascade has yet to be determined. Therefore, this study aimed to explore the regulatory mechanism of TsI in modulating apoptosis in SIONFH through the PI3K/AKT/mTOR pathway, with the goal of identifying novel therapeutic strategies for this condition.

## 2 Materials and Methods

### 2.1 Ethics Statement

All animal procedures were reviewed and approved by the Animal Ethics Committee of Yantai Shan Hospital (Approval No. 2025‐221). Experiments were conducted in strict accordance with institutional and national guidelines for the care and use of laboratory animals. All efforts were made to minimize animal suffering, including the use of heating pads, sterile surgical techniques, and administration of physiological saline to reduce procedural discomfort.

### 2.2 Establishment of the SIONFH Rat Model

Thirty‐six specific pathogen free (SPF) male Sprague–Dawley rats (12 weeks old, weighing 420 ± 20 g) were obtained from Hunan Slaike Jingda Laboratory Animal Co. Ltd. (Hunan, Changsha, China). Rats were housed under standard SPF conditions (24°C ± 2°C, 50% ± 10% humidity, 12 h light–dark cycle) with ad libitum access to food and water.

To induce SIONFH, rats received intraperitoneal injections of LPS (20 μg/kg/d; V60063, Invivochem, Libertyville, IL, USA) for two consecutive days, followed by intramuscular injections of methylprednisolone (MPS; 40 mg/kg/d; 83‐43‐2, Invivochem) for three consecutive days. Control rats (Normal group) received equal volumes of 0.9% saline [11].

After a one‐week acclimation period, rats were randomly divided into six groups (*n* = 6 per group), including the Normal group (untreated control rats), Model group (SIONFH rats without treatment), TsI group [beginning 4 weeks after the MPS induction, rats received daily intraperitoneal injections of TsI (10 mg/kg; CAS: 568‐73‐0, Aladdin, Beijing, China) dissolved in 1% dimethyl sulfoxide (DMSO) for 4 consecutive weeks [11]], Vehicle group [SIONFH rats received 1% DMSO (vehicle for TsI) intraperitoneally for 4 weeks], TsI + 740 Y‐P group [rats received both TsI and the PI3K activator 740 Y‐P (10 mg/kg; CAS: 1236188‐16‐1, Aladdin) dissolved in 90% corn oil and 10% DMSO for 4 weeks [22]], and TsI + Vehicle group [rats were treated with TsI and a corresponding volume of 740 Y‐P vehicle (90% corn oil and 10% DMSO) for 4 weeks].

At the end of the 4‐week treatment, all rats were euthanized via intraperitoneal injection of sodium pentobarbital (200 mg/kg; P3761, Sigma‐Aldrich, St. Louis, MO, USA). Bilateral femoral heads were harvested from 5 mm below the joint surface. The left femoral heads were reserved for micro‐computed tomography (microCT) and Western blot analysis, while the right femoral heads were processed for histological evaluations, including hematoxylin and eosin (H&E) staining, immunohistochemistry (IHC), and terminal deoxynucleotidyl transferase dUTP nick‐end labeling (TUNEL) staining.

### 2.3 MicroCT Scan and Quantitative Analysis

MicroCT scans of the left femoral heads were performed using a Quantum GX2 micro‐CT scanner (PerkinElmer, Waltham, MA, USA) at 90 kV/88 μA, with a 5 mm scan length and a resolution of 10 μm/pixel. Structural parameters, including bone mineral density (BMD), trabecular number (Tb.N), bone volume/total volume (BV/TV), trabecular thickness (Tb.Th), and trabecular separation (Tb.Sp), were quantitatively analyzed.

### 2.4 Morphology Evaluation

Histological analysis was performed using H&E staining. Briefly, longitudinal femoral head specimens were fixed in 10% neutral buffered formalin, decalcified with ethylene diamine tetra‐acetic acid (CAS: 60‐00‐4, Sigma‐Aldrich), embedded in paraffin, and sectioned at 4 μm thickness. Sections were deparaffinized in xylene, rehydrated through ethanol, and stained with hematoxylin for 10 min. After rinsing with running water, differentiation was conducted using 0.7% hydrochloric acid ethanol for a few seconds. The sections were then blued under running water for approximately 15 min, counterstained with eosin in 95% ethanol for 30 s, dehydrated, cleared, and mounted using neutral resin. Morphological changes were observed using a light microscope (Olympus, Tokyo, Japan). The rate of empty lacunae was calculated in 10 randomly selected fields (200 × magnification) per section, counting 20 bone lacunae per field. The percentage of empty lacunae was determined as [11]:

$$\text{Empty lacunae rate}\;\left(\%\right)=\left(\text{Number of empty lacunae}/\text{Total lacunae}\right)\times 100$$

### 2.5 IHC

For immunohistochemical detection, longitudinal femoral head sections (5 μm) were decalcified, paraffin‐embedded, and rehydrated. Antigen retrieval was performed in a citrate buffer (8.2% sodium citrate and 1.8% citric acid) for 10 min, followed by blocking of endogenous peroxidase with 3% hydrogen peroxide for 15 min. After blocking with goat serum (SL038, Solarbio, Beijing, China) for 15 min, sections were incubated overnight at 4°C with rabbit anti‐Osteopontin antibody (1:2000, ab214050, Abcam, Cambridge, MA, USA). Subsequently, sections were incubated with HRP‐conjugated goat anti‐rabbit IgG secondary antibody (1:1000, ab214050, Abcam) at 37°C for 60 min. Color development was performed with diaminobenzidine tetrahydrochloride (DA1016, Solarbio) and sections were counterstained with hematoxylin (H8070, Solarbio). Images were captured using an Olympus microscope, and quantitative analysis of IHC staining was conducted using Image Pro Plus 6.0 software (Media Cybernetics, Silver Spring, MD, USA).

### 2.6 TUNEL And Osteopontin Fluorescence Dual Labeling

To evaluate apoptosis, femoral head tissue sections were subjected to TUNEL using a commercial kit (12156792910, Roche, Indianapolis, IN, USA). After deparaffinization and rehydration, TUNEL staining was conducted following the manufacturer‘s instructions. Sections were washed three times with tris‐buffered saline (TBS; 5 min each), followed by incubation with anti‐Osteopontin antibody (1:50, ab214050, Abcam) overnight. After additional TBS washes, sections were incubated with Alexa Fluor 488‐conjugated goat anti‐rabbit IgG secondary antibody (1:200, ab150077, Abcam). Apoptotic cells were visualized as green fluorescence using a fluorescence microscope (Zeiss, Oberkochen, BW, Germany). Five non‐overlapping fields (top, bottom, left, right, center) were selected per section, and the percentage of TUNEL‐positive cells was counted and averaged across fields.

### 2.7 Western Blot Analysis

The gathered femoral head tissues were processed using radioimmunoprecipitation assay lysis buffer (AR0107, Boster Biological Technology, Wuhan, Hubei, China), and protein concentrations were determined using a bicinchoninic acid protein assay kit (AR1189, Boster Biological Technology). Equal amounts of protein were denatured by boiling in loading buffer for 5 min and separated via sodium dodecyl sulfate‐polyacrylamide gel electrophoresis. Proteins were then transferred to polyvinylidene fluoride membranes and blocked in 3% bovine serum albumin (BSA) for 2 h at room temperature.

Membranes were incubated overnight at 4°C with the following primary antibodies: anti‐Bax (1:2000, ab32503, Abcam), anti‐Bcl‐2 (1:2000, ab194583, Abcam), anti‐cleaved‐caspase‐3 (1:500, ab32042, Abcam), anti‐p‐AKT (1:500, ab8805, Abcam), anti‐p‐PI3K (1:1000, ab182651, Abcam), anti‐AKT (1:1000, ab38449, Abcam), anti‐PI3K (1:1000, ab191606, Abcam), anti‐mTOR (1:10000, ab134903, Abcam), and anti‐p‐mTOR (1:1000, ab109268 Abcam). β‐Actin (1:1000, ab8227, Abcam) was used as the internal loading control. Following primary antibody incubation, membranes were incubated with horseradish peroxidase‐conjugated goat anti‐rabbit IgG (1:2000, ab205718, Abcam) for 1 h at room temperature in the dark.

Signal detection was performed using an enhanced chemiluminescence (ECL) detection reagent (AR1191, Boster Biological Technology). Band intensities were quantified using Image Pro Plus 6.0 software (Media Cybernetics, Bethesda, MD, USA) and protein expression was normalized to β‐actin. Each experiment was conducted in triplicate to ensure reproducibility.

### 2.8 Statistical Analysis

All data were analyzed using GraphPad Prism 8.01 (GraphPad Software, San Diego, CA, USA). The Kolmogorov–Smirnov test was employed to assess the normality of continuous variables. Normally distributed data were expressed as mean ± standard deviation. For comparisons between two groups, an independent sample *t*‐test was used, while comparisons among multiple groups were analyzed using one‐way analysis of variance (ANOVA) followed by Tukey's post hoc test. *p* < 0.05 was considered statistically significant.

## 3 Results

### 3.1 TsI Attenuates Bone Loss and Mitigates SIONFH in Rats

**FIGURE 1:**
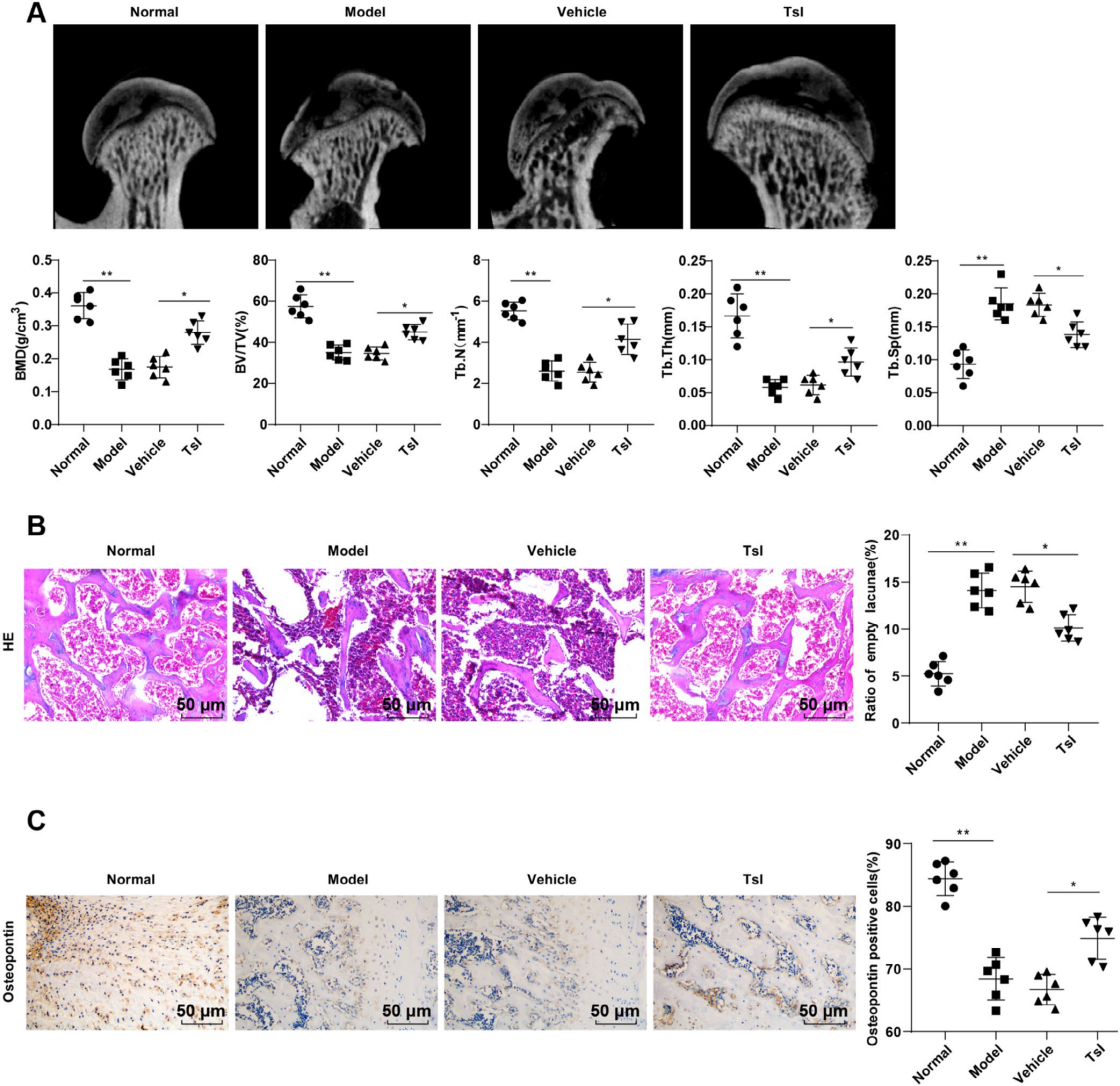
TsI reduced bone loss and attenuated SIONFH in rats. (A) microCT to determine BMD, BV/TV, Tb.N, Tb.Th, and Tb.Sp. (B) H&E staining to count empty cavities. (C) IHC to assess Osteopontin (osteoblast markers) in the femoral head. *n* = 6. Data were represented as mean ± standard deviation. One‐way ANOVA was utilized for data comparisons among multiple groups, with Tukey's test used for post hoc test. *p < 0.05, **p < 0.01.

To investigate the therapeutic potential of TsI in SIONFH, an in vivo rat model was established using LPS and MPS, as described previously [11]. Micro‐CT analysis revealed significantly decreased levels of BMD, BV/TV, Tb.N, and Tb.Th, along with a notable increase in Tb.Sp in the Model group compared to the Normal group (all *p* < 0.01, Figure 1A). Histological examination via H&E staining showed a marked increase in the rate of empty lacunae in the Model group versus the Normal group (*p* < 0.01, Figure 1B). Immunohistochemical analysis further demonstrated a significant reduction in Osteopontin‐positive cells in the Model group (*p* < 0.01, Figure 1C).

Following TsI administration initiated 4 weeks after the final MPS injection, rats in the TsI‐treated group exhibited substantial recovery of bone parameters, as evidenced by increased BMD, BV/TV, Tb.N, and Tb.Th, along with a decrease in Tb.Sp (all *p* < 0.05 vs. Vehicle, Figure 1A). Correspondingly, TsI significantly reduced the percentage of empty lacunae (*p* < 0.05, Figure 1B) and restored Osteopontin expression in the femoral head (*p* < 0.05, Figure 1C). These data suggest that TsI effectively alleviates bone loss and improves femoral head microarchitecture in SIONFH rats.

### 3.2 TsI Suppresses Cell Apoptosis in SIONFH

**FIGURE 2:**
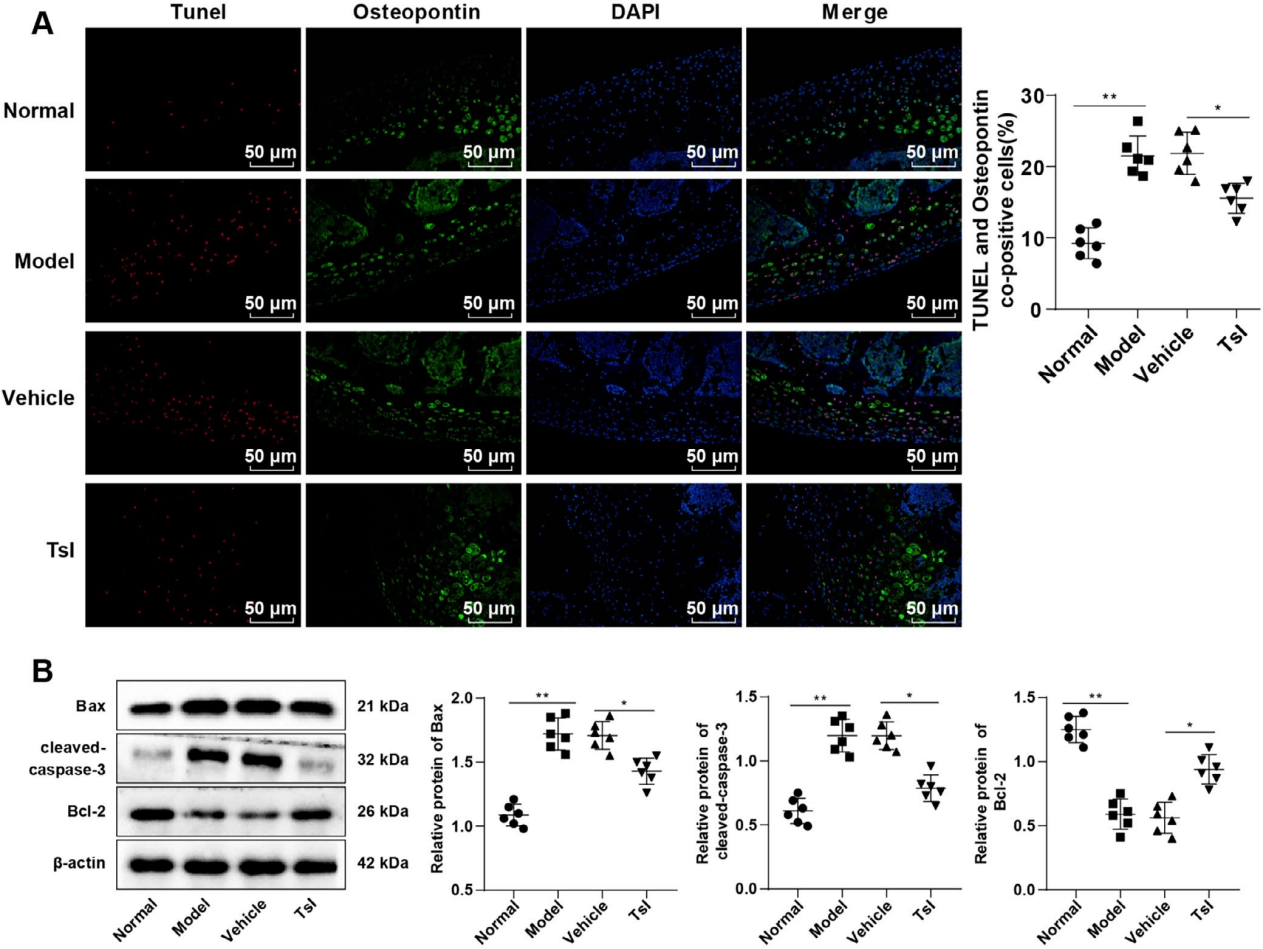
TsI lessened cell apoptosis in rats with SIONFH. (A) Cell apoptosis was evaluated by TUNEL and Osteopontin fluorescence dual labeling. (B) Levels of pro‐apoptotic proteins (Bax, cleaved‐caspase 3) and anti‐apoptotic protein (Bcl‐2) were measured by Western blot. *n* = 6. Data were expressed as mean ± standard deviation. Data comparisons among groups were performed with the aid of one‐way ANOVA, and post hoc tests were implemented by Tukey's multiple comparison test. *p < 0.05, **p < 0.01.

To determine whether TsI exerts its protective effect by modulating cell apoptosis, TUNEL and Osteopontin dual labeling assays were performed on femoral head tissues. The Model group exhibited a significantly elevated number of TUNEL and Osteopontin co‐positive cells compared to the Normal group, whereas TsI treatment markedly reduced this apoptotic index compared to the Vehicle group (all *p* < 0.05, Figure 2A).

Western blot analysis revealed that the expression levels of pro‐apoptotic markers Bax and cleaved‐caspase‐3 were significantly elevated in the Model group (*p* < 0.01 vs. Normal), while the anti‐apoptotic protein Bcl‐2 was significantly reduced. Notably, TsI administration led to decreased expression of Bax and cleaved‐caspase‐3 and increased expression of Bcl‐2 compared to the Vehicle group (all *p* < 0.05, Figure 2A,B). Collectively, these findings confirm that TsI reduces cell apoptosis in the femoral head and contributes to the amelioration of SIONFH pathology.

### 3.3 TsI Inhibits Activation of the PI3K/AKT/mTOR Pathway in Femoral Head Tissues of SIONFH Rats

**FIGURE 3:**
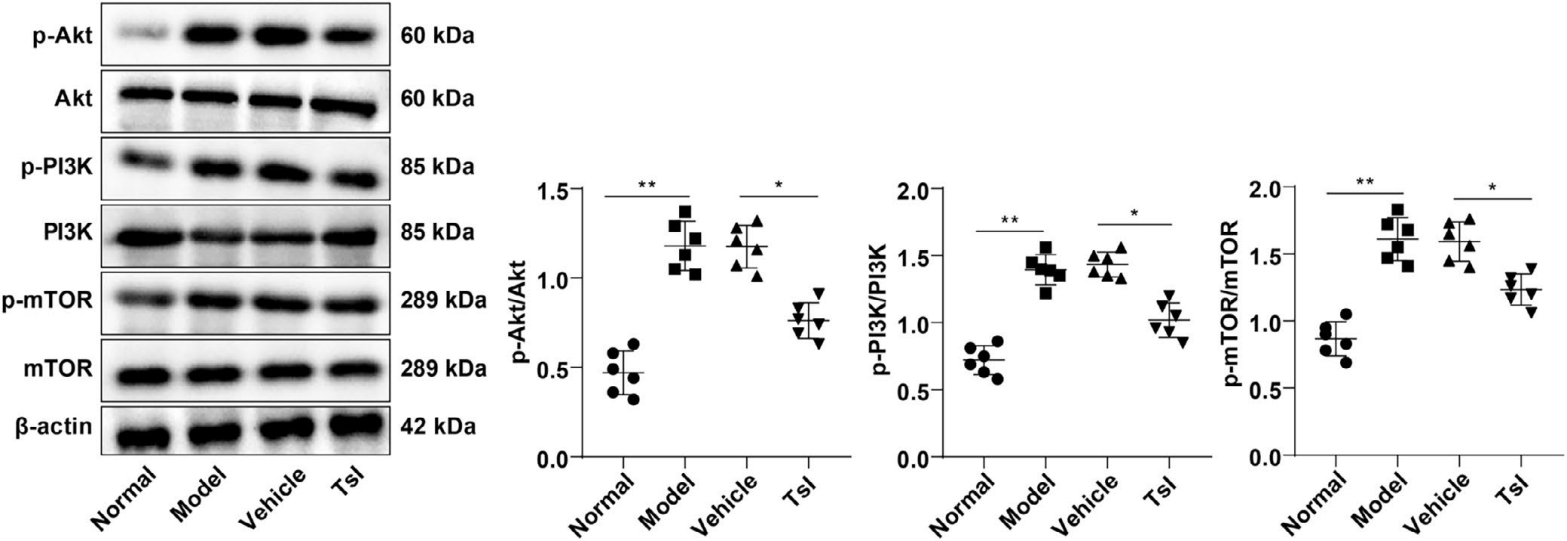
TsI repressed the PI3K/AKT/mTOR pathway activation in femoral head tissues of rats with SIONFH. Western blot to determine the phosphorylation levels of AKT, PI3K, and mTOR. *n* = 6. Data were indicated as mean ± standard deviation. One‐way ANOVA was used to compare the data among multiple groups, followed by Tukey's test. *p < 0.05, **p < 0.01.

To explore the potential regulatory mechanism of TsI, we examined whether it affects the activation of the PI3K/AKT/mTOR signaling pathway in SIONFH. Western blot analysis revealed that phosphorylation levels of AKT, PI3K, and mTOR were significantly elevated in the Model group compared to the Normal group (all *p* < 0.01), indicating pathway activation during disease progression. However, TsI treatment markedly suppressed the phosphorylation of PI3K, AKT, and mTOR compared to the Vehicle group (*p* < 0.05, Figure 3), suggesting that TsI effectively attenuates PI3K/AKT/mTOR pathway activation in the femoral head tissues of SIONFH rats.

### 3.4 PI3K/AKT/mTOR Pathway Activation Attenuates the Effects of TsI Against MPS‐Induced ONFH and Apoptosis

**FIGURE 4:**
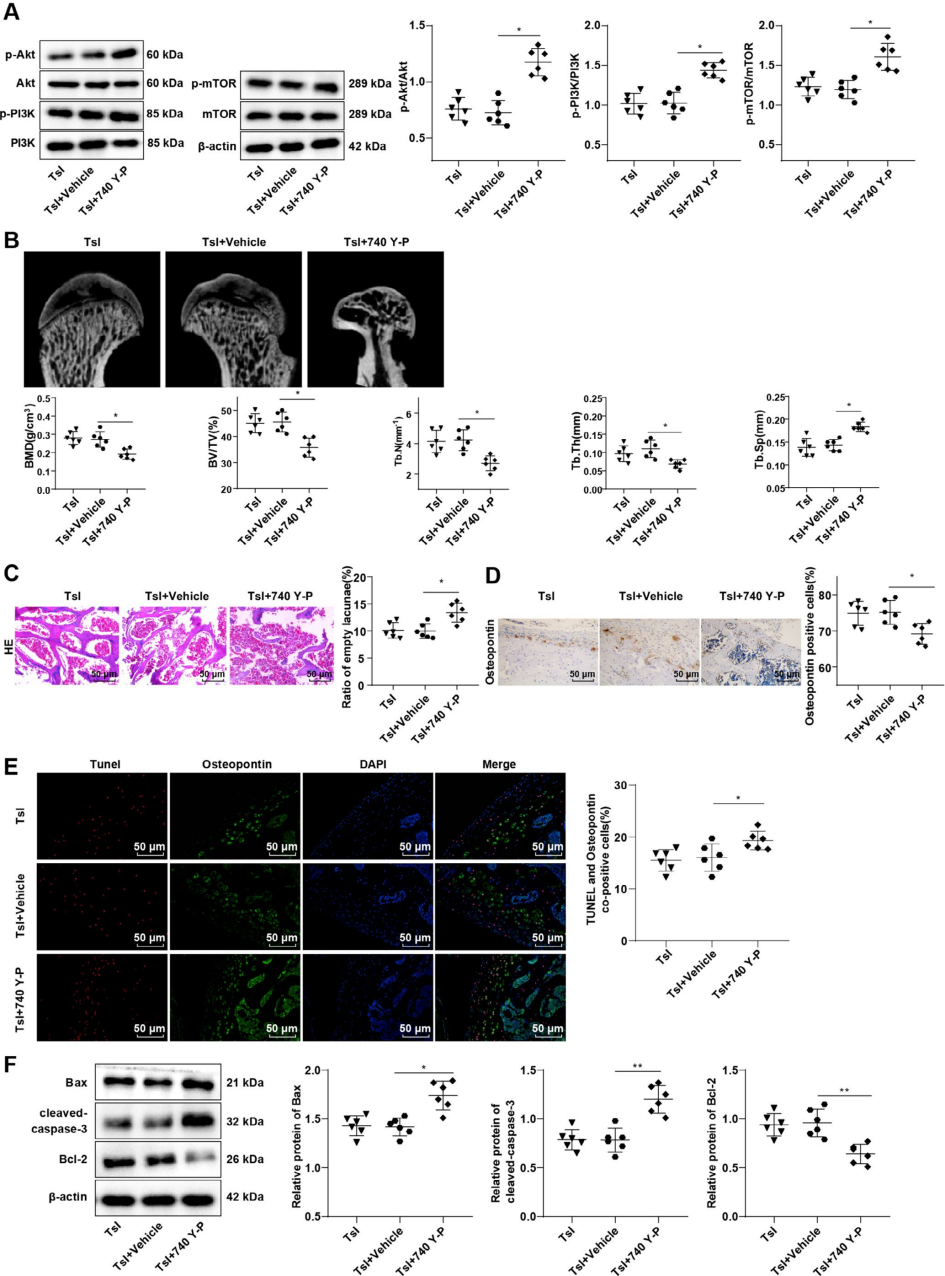
Activation of the PI3K/AKT/mTOR pathway partially annulled the mitigative roles of TsI in ONFH and osteoblast apoptosis provoked by MPS in rats. (A) The phosphorylation levels of AKT, PI3K and mTOR were assayed by Western blot. (B) BMD, BV/TV, Tb.N, Tb.Th, and Tb.Sp were assessed by microCT. (C) H&E staining to count empty cavities. (D) Osteopontin (osteoblast marker) in the femoral head was detected by IHC. (E) TUNEL and Osteopontin fluorescence dual labeling to evaluate osteoblast apoptosis. (F) Anti‐apoptotic protein (Bcl‐2) and pro‐apoptotic protein (Bax, cleaved‐caspase 3) levels were measured by Western blot. *n* = 6. Data were expressed as mean ± standard deviation, with one‐way ANOVA exploited to compare data among groups, and Tukey's test used for post hoc test. *p < 0.05, **p < 0.01.

To validate the involvement of the PI3K/AKT/mTOR pathway in TsI‐mediated protection, rats were co‐treated with TsI and the PI3K activator 740 Y‐P. Compared to the TsI + Vehicle group, rats in the TsI + 740 Y‐P group showed significant upregulation in phosphorylated AKT, PI3K, and mTOR levels in the femoral head (all *p* < 0.05), confirming successful pathway activation (Figure 4A).

Functionally, pathway activation resulted in significantly reduced BMD, BV/TV, Tb.N, and Tb.Th, along with increased Tb.Sp (all *p* < 0.05, Figure 4B), indicating reversal of TsI's bone‐protective effects. H&E staining demonstrated an elevated rate of empty lacunae (*p* < 0.05, Figure 4C), while IHC showed fewer Osteopontin‐positive cells in the TsI + 740 Y‐P group (*p* < 0.05, Figure 4D). Furthermore, dual TUNEL and Osteopontin staining revealed an increased proportion of apoptotic osteocytes (*p* < 0.05, Figure 4E), accompanied by higher expression of Bax and cleaved‐caspase‐3 and reduced Bcl‐2 levels (*p* < 0.05, Figure 4F).

Collectively, these findings confirm that PI3K/AKT/mTOR pathway activation partially reverses the anti‐apoptotic and osteoprotective effects of TsI, reinforcing the role of this pathway in the pathogenesis of SIONFH and in the therapeutic action of TsI.

## 4 Discussion

ONFH is widely recognized as a debilitating condition that imposes substantial personal and socioeconomic burdens worldwide [23]. Epidemiological studies have indicated that non‐traumatic ONFH predominantly affects relatively young adults, particularly men aged 20–50 years [24]. As the disease progresses, structural deterioration of the femoral head often culminates in secondary hip arthritis, necessitating surgical intervention in advanced stages [25]. In light of prior findings suggesting that TsI has therapeutic potential in SIONFH [5], the present study provides mechanistic insights into its function. Specifically, we demonstrate that TsI alleviates SIONFH in rats by suppressing the activation of the PI3K/AKT/mTOR signaling pathway, thereby reducing apoptosis, offering a promising molecular target for SIONFH therapy.

Flavonoids have attracted increasing attention in the context of ONFH due to their anti‐osteoporotic and cytoprotective properties. Compounds such as luteolin [26], icariin [27], and naringin [28] have shown notable efficacy. Derived from *S. miltiorrhiza* Bunge, TsI is a major bioactive diterpenoid quinone that has previously demonstrated anti‐osteoclastic properties in vitro [7, 29]. Beyond its bone‐related effects, TsI is known for its antioxidant, antitumor, and anti‐inflammatory functions [30]. For example, Wang et al. reported that TsI exerts neuroprotective effects by downregulating pro‐inflammatory genes in LPS‐stimulated microglia [31], and its anti‐inflammatory properties have been validated in adjuvant‐induced arthritis models [32]. Nevertheless, the protective mechanism of TsI in ONFH remains insufficiently understood.

Our data reveal that TsI‐treated SIONFH rats exhibit significantly improved femoral head bone parameters, including increases in BV/TV, BMD, Tb.Th, and Tb.N, alongside reduced Tb.Sp and a lower rate of empty lacunae. TsI also restored Osteopontin expression, indicative of improved bone remodeling and osteocyte survival. These structural improvements are consistent with previous findings that other derivatives of *S. miltiorrhiza* , such as Tanshinone IIA, promote osteogenesis via the extracellular signal‐regulated kinases 1/2‐dependent runt‐related transcription factor 2 (ERK1/2‐Runx2) signaling pathway [33]. In addition, TsI has been shown to mitigate bone loss and enhance angiogenesis in SIONFH rats, while preserving the angiogenic capacity of endothelial cells and protecting them from steroid‐induced apoptosis [11]. Similarly, TsI has been reported to inhibit osteoclast formation and stimulate osteogenesis in SIONFH [5]. Collectively, our findings further substantiate the osteoprotective and anti‐apoptotic roles of TsI in SIONFH pathophysiology.

TsI has previously been shown to alleviate chondrocyte apoptosis, extracellular matrix degradation, and inflammatory responses in OA models, thereby mitigating disease progression in mice [12]. To assess the effect of TsI on cell apoptosis in the context of SIONFH, our study demonstrated that model rats exhibited a significant increase in TUNEL and Osteopontin co‐positive cells, as well as elevated levels of Bax and cleaved‐caspase‐3 proteins, along with reduced Bcl‐2 expression, indicators of heightened apoptosis. Conversely, TsI treatment reversed these alterations, suggesting its robust anti‐apoptotic effects. These findings are in line with previous reports that Tanshinone IIA mitigates dexamethasone‐induced osteoblast apoptosis by inhibiting reactive oxygen species accumulation [34]. More broadly, the Tanshinone family has been implicated in regulating bone remodeling by promoting osteoblastogenesis and restraining both osteoclast activity and osteoblast apoptosis [35]. Together, our data underscore that TsI effectively suppresses apoptosis in SIONFH rats.

Importantly, the PI3K/AKT/mTOR signaling pathway has emerged as a critical axis in glucocorticoid‐induced apoptosis. Previous studies have shown that pinocembrin attenuates glucocorticoid‐induced apoptosis via inhibition of this pathway [19], and TsI has also been reported to inactivate PI3K/AKT/mTOR signaling [21]. In accordance, our results revealed that TsI significantly reduced phosphorylation levels of AKT, PI3K, and mTOR in femoral head tissues, confirming pathway inactivation. Supporting this, TsI has been shown to inhibit the proliferation of vascular smooth muscle cells by suppressing IGF‐1R/PI3K signaling [36]. Likewise, Tanshinone IIA has been reported to alleviate osteoclastogenesis in ovariectomized mice by dampening NF‐kB and AKT signaling pathways [37], and also to inhibit PI3K/AKT/mTOR signaling in endometriosis by modulating angiogenesis, invasion, and adhesion [38].

Critically, when the PI3K/AKT/mTOR pathway was pharmacologically reactivated using the PI3K activator 740 Y‐P, the protective effects of TsI on bone structure and apoptosis were partially reversed. Activation of this pathway led to decreased levels of Osteopontin expression, BMD, BV/TV, Tb.N, Tb.Th, and Bcl‐2, alongside increased Tb.Sp, empty lacunae rate, TUNEL, and Osteopontin co‐positive cells, as well as upregulated Bax and cleaved‐caspase‐3. These findings further confirm that the therapeutic effects of TsI are, at least in part, mediated through the suppression of PI3K/AKT/mTOR pathway activation in the context of steroid‐induced osteonecrosis.

From the clinical perspective, TsI presents promising advantages as a potential therapeutic agent for SIONFH. As a bioactive compound extracted from the traditional medicinal herb *S. miltiorrhiza* , TsI exhibits a favorable safety profile and is generally well tolerated, offering a natural alternative with reduced toxicity compared to certain conventional chemotherapeutics [39]. Given the chronic nature of SIONFH, especially in young and middle‐aged populations, the potential for long‐term use of TsI with fewer adverse effects is clinically significant [40]. Moreover, validating TsI as an effective treatment for SIONFH could provide a non‐surgical intervention strategy, potentially minimizing surgical risks and complications while alleviating economic burdens associated with surgical procedures and post‐operative recovery.

However, several challenges remain before TsI can be routinely translated into clinical practice. First, the precise molecular mechanisms underlying its action in SIONFH require further clarification, particularly concerning specific downstream targets and interacting signaling pathways. Second, large‐scale, randomized clinical trials are essential to rigorously evaluate the safety, therapeutic efficacy, optimal dosing, and administration routes of TsI. Additionally, future research should explore the potential synergistic effects of combining TsI with other therapeutic protocols or other pharmacologic agents to enhance overall treatment outcomes.

In conclusion, this study demonstrated that TsI attenuates cell apoptosis in SIONFH by inhibiting the PI3K/AKT/mTOR signaling pathway, supporting its potential as a therapeutic agent and providing mechanistic insight into its mode of action. Nonetheless, this work was limited to preclinical investigation, and clinical validation remains necessary. Future research will aim to investigate additional molecular pathways regulated by TsI, such as the circ_0000376/miR‐432‐5p/BCL2, KDM4D/p53, and NLRP3 inflammasome pathways, to comprehensively elucidate its therapeutic mechanisms and optimize its translational potential.

## Ethics Statement

All animal procedures were reviewed and approved by the Animal Ethics Committee of Yantai Shan Hospital (Approval No. 2025‐221). Experiments were conducted in strict accordance with institutional and national guidelines for the care and use of laboratory animals. All efforts were made to minimize animal suffering, including the use of heating pads, sterile surgical techniques, and administration of physiological saline to reduce procedural discomfort.

## Conflicts of Interest

The authors declare no conflicts of interest.

## Data Availability

The data that support the findings of this study are available from the corresponding author upon reasonable request.
